# Distinct allelic expression patterns of imprinted *IGF2* in adenocarcinoma and squamous cell carcinoma of the lung

**DOI:** 10.3892/ol.2014.2572

**Published:** 2014-09-29

**Authors:** SATORU OZAKI, EI KAWAHARA, SHIORI MAENAKA, NGUYEN VIET HOANG, TAKERU OYAMA, MIWA IMAI, MAKOTO ODA, SEIJI YANO

**Affiliations:** 1 Department of Clinical Laboratory Science, Kanazawa University, Ishikawa 920-0942, Japan; 2 Department of Pathology, Kanazawa University, Ishikawa 920-0942, Japan; 3 Department of Health Science, Ishikawa Prefectural Nursing University, Ishikawa 929-1212, Japan; 4 Department of Lung Surgery, Kanazawa University Hospital, Ishikawa 920-8641, Japan; 5 Department of Internal Medicine, Kanazawa University Cancer Research Institute, Ishikawa 920-1192, Japan

**Keywords:** genomic imprinting, insulin-like growth factor 2, lung cancer, adenocarcinoma, microdissection

## Abstract

The insulin-like growth factor 2 gene (*IGF2*) is an imprinting gene, which mediates cell growth and apoptosis. The loss of imprinting (LOI) of *IGF2* has been associated with the development of cancer. In the present study, loss LOI of *IGF2* in lung cancer was analyzed using polymerase chain reaction-restriction fragment length polymorphism (PCR-RFLP) in combination with DNA sequencing of samples collected by laser capture microdissection. The status of each sample was assigned as imprinting when PCR-RFLP revealed only one band or sequence with a single peak; otherwise, the case was classified as LOI. LOI was identified in eight out of 13 adenocarcinoma cases (62%), but was not detected in any of the nine squamous cell carcinoma cases (0%). These results suggest that *IGF2* LOI is involved in the molecular pathogenesis of lung adenocarcinoma, but not squamous cell carcinoma, and that LOI may be detected through increased *IGF2* expression levels.

## Introduction

Insulin-like growth factor 2 (*IGF2*) is an important mediator of cell growth and apoptosis that is paternally expressed. *IGF2* loss of imprinting (LOI) is predominantly indicated by the activation of the usually silenced maternal allele, with subsequent expression of the two gene copies (1). This is associated with a number of hereditary overgrowth conditions, including Beckwith-Wiedemann syndrome (2) and cancer that exhibits cell overgrowth resulting from *IGF2* overexpression (3). LOI has been reported in a variety of cancer types, in particular colorectal carcinoma (4–6). However, to the best of our knowledge, only one study examining LOI in lung carcinoma (7) has been published; LOI was detected in 47% adenocarcinomas, although the prevalence of LOI in squamous cell carcinomas was not mentioned. However, other studies have found the prevalence of LOI in squamous cell carcinomas and urothelial carcinomas in other organs to be relatively low. For example, the prevalence was found to be 21% in esophageal cancer (8) and 22% in bladder cancer (9). By contrast, the prevalence of LOI was 44–54% (4,7) and 49% (10) in colorectal adenocarcinoma and gastric carcinoma, respectively. However, considerable variation has been reported.

Personalized cancer therapy has been applied to lung adenocarcinoma patients through the development of molecular-targeted therapeutic drugs against driver oncogenes. For example, lung adenocarcinoma patients with an epidermal growth factor receptor (*EGFR*) mutation or with an *ELM4*-*ALK* fusion protein have been shown to respond well to the corresponding drugs (11). Similarly, molecular-targeted therapy for insulin growth factors (IGFs) has been developed for a variety of cancer types, including non-small cell lung cancer (12,13). Therapeutic methods that target the IGF1 receptor (IGF1R) have achieved certain success, although a modified therapy that targets and insulin receptor (IR) has proved more effective (13). IGF1 and IR are receptors for IGF2 that induce signal transduction resulting in cell growth; however, the IGF2 receptor interrupts IGF2 signal induction (13). Silencing of the *IGF2* gene was recently reported to result in apoptosis only for *IGF2* LOI colorectal carcinomas (14). Thus, *IGF2* LOI lung carcinoma appears to be a good candidate for molecular-targeted therapy.

To examine *IGF2* LOI, polymerase chain reaction-restriction fragment length polymorphism (PCR-RFLP) has been previously employed, but precise analysis is hampered by a number of problems, including lymphocyte contamination and heteroduplex formation during PCR (15). In the present study, the precise incidence of *IGF2* LOI in lung carcinomas was examined using PCR-RFLP in combination with DNA sequencing of samples obtained by a laser capture microdissection (LCM) method, as reported previously (16).

## Materials and methods

### Materials

Tissue samples were obtained from 32 patients with lung cancer (19 with adenocarcinoma, 12 with squamous cell carcinoma and one with large cell carcinoma). Sections of carcinoma tissues and non-tumor lung tissue were removed and frozen immediately following surgery at Kanazawa University Cancer Research Hospital (Kanazawa, Japan). The tissues were stored at −80°C until further analysis.

### LCM

Frozen 10-μm tissue sections were fixed with 70% ethanol for 10 min and stained with Kernechtrot (Merck, Darmstadt, Germany). A total of ~1,000 carcinoma cells in the frozen sections were punched out using a LCM system (LM100; Olympus Corporation, Tokyo, Japan) and collected on a plastic cap (CapSureTM LCM Caps; Arcturus, Mountain View, CA, USA). All specimens were assessed histologically by a pathologist (Professor E. Kawahara).

### PCR-RFLP and direct sequencing

Genomic DNA from the frozen sections of the normal tissues was extracted using a Wizard^®^ SV Genomic DNA Purification system (Promega Corporation, Fitchburg, WI, USA). To extract total RNA from the microdissected carcinoma cells, a PicoPure™ RNA isolation kit (Arcturus) was used. The extracted RNA was further purified using the acid guanidinium phenol chloroform method, and possible contaminating DNAs were digested with DNase I (Takara, Tokyo, Japan). Aliquots of 1 μl extracted total RNA in a volume of 20 μl were converted to cDNA using AMV reverse transcriptase (Promega Corporation). The reverse transcription reaction was performed for 30 min at 42°C and then the sample was heated for 5 min at 99°C to inactivate the enzyme. Genomic DNA from normal whole tissues was amplified by PCR using the following primer pair for exon 9 of human *IGF2*: Forward, 5′-CTTGGACTTGAGTCAAATTGG-3′ and reverse, 5′-GGTCGTGCCAATTACATTTCA-3′. The tumor cDNAs obtained from the microdissection samples were amplified by nested PCR using the same primer pair followed by the second primer pair: Forward, 5′-CTTGGACTTTGAGTCAAATTGG-3′ and reverse, 5′-GGTTTTCATGCTCTGTCCTC-3′. The amplified 292 and 257 base-pair fragments were digested by *Apa*I (Toyobo, Osaka, Japan), which recognizes the C/T single nucleotide polymorphism site in the amplified area. Successful digestion of contaminating DNA was confirmed by PCR designed to amplify the exon-intron spanning region of the integrin β5 gene. Analysis of the polymorphism in the *IGF2* gene was then performed. The digested and undigested DNAs were run on 2% agarose gels containing ethidium bromide. The gel revealed three bands for heterozygotes, in which the restriction enzyme recognition sequence was present in one allele and absent in the other. The PCR products were also sequenced using Dye Terminator 1.1 (Applied Biosystems, Carlsbad, CA, USA) and ABI PRISM^®^ 3130×l Genetic Analyzer (Applied Biosystems).

### Determination of imprinting status

Digital images of the bands scanned into a computer were analyzed using the Image J software (National Institutes of Health, Bethesda, MD, USA). The band densities were measured and the ratio of the two bands was calculated. Similarly, the ratio of the two C and T peaks was calculated. The imprinting status was determined according to the criterion described in our previous study (10). In the case that two clear bands were detected with PCR-RFLP (ratio = 10–90%) and the sequence pattern revealed two clear peaks (ratio = 20–80%), the status was considered to be LOI; otherwise, the status was considered as imprinted.

### Statistical analysis

Statistical analyses were performed using StatMate software version 4.0 (ATMS, Co., Ltd., Tokyo, Japan) to perform the χ^2^ test. P<0.05 was considered to indicate a statistically significant difference.

### Ethics

The study was approved by the Instititional Review Board for Analytical Research for Human Genome and Gene at Kanazawa University. All patients provided informed consent prior to sample collection according to the institutional guidelines.

## Results

### LOI and imprinting status of lung carcinoma samples

**Figure 1:**
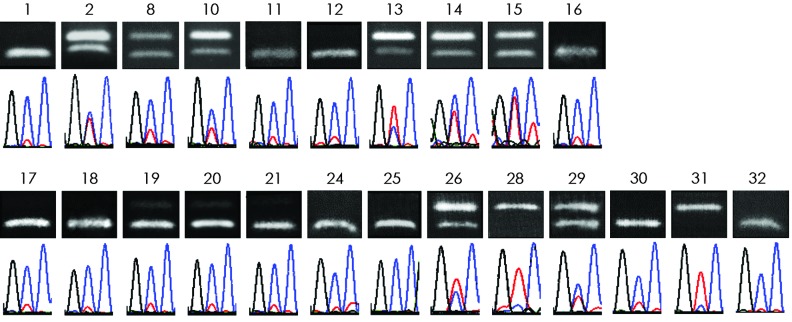
Polymerase chain reaction (PCR)-restriction fragment length polymorphism analysis using IGF2 gene *Apa*I polymorphism and sequencing for laser-capture-microdissected samples of lung carcinomas. The PCR products were digested with *Apa*I and run on 2% agarose gels. In the electropherogram, green indicates adenine; black, guanine; blue, cytosine and red, thymine.

The genomic DNA of whole non-tumor mucosa was employed to screen for whether the samples were informative or not, using the PCR-based, *IGF2* gene *Apa*I polymorphism. Of the 32 lung cancer cases, 23 (72%) were informative. All informative cases were examined for imprinting status in the microdissected carcinoma cells. The PCR products from the cDNA of the carcinoma cells revealed either two bands or a single band following *Apa*I analysis, and either two C and T peaks or one peak with a possible background peak subsequent to sequence analysis (Fig. 1). Prior to determining whether the status was imprinted or LOI, the density ratio between the top and bottom bands, determined using PCR-RFLP, and the height ratio of the C and T peaks, examined using sequence analysis, were measured and calculated (Table I). Each case was identified as either imprinted or LOI, according to the criteria described above (Table I). LOI was detected in the adenocarcinoma and large cell carcinoma specimens, but was not detected in the squamous cell carcinoma samples. The percentage of LOI was 39% in total lung carcinomas (9 out of 23 cases) and 62% (8 out of 13 cases) in adenocarcinomas.

### Association between LOI and histological grade

Since a previous study concerning *IGF2* LOI in lung adenocarcinoma refers to histological type (7), the association between the histological grade of the adenocarcinoma sample and LOI was also analyzed in the present study. Four cases of well-differentiated adenocarcinoma were found to be LOI and one case of poorly differentiated adenocarcinoma was imprinted (Table I). These results do not support the previous suggestion that *IGF2* LOI occurs more frequently in poorly differentiated adenocarcinomas (7). Statistical analysis revealed no significant association between the degree of differentiation and LOI.

## Discussion

In the present study, the status of genomic imprinting of IGF2 in lung cancer was evaluated. IGF2 LOI was found to increase IGF2 signaling by increasing the proliferation of expression-related genes (17). We hypothesize that IGF2 LOI leads to an increased the risk of malignant transformation.

In this study, *IGF2* LOI was detected in half the cases of adenocarcinoma, but not in any cases of lung squamous cell carcinoma, which suggests an association between *IGF2* LOI and the histological type of the tumor. A number of adenocarcinoma-specific gene alterations are known, including *EGFR*, *KRAS* and *BRAF*, and these affect the gene products of the MAP kinase and PI3/AKT signaling pathways (18,19). IGF2 binds to IR or IGF1R, thereby activating the same pathways (12). In this context, the effect of *IGF2* overexpression appears comparable with the effect of other gene alterations specific to adenocarcinoma.

The incidence of *IGF2* LOI in adenocarcinoma (62%) appears high as compared with other adenocarcinoma-specific gene alterations. However, the incidence of gene alteration may reflect population differences. A previous study indicated that the most frequent gene alteration in lung adenocarcinomas in Japanese patients is the *EGFR* mutation, with an incidence of 38%, while the most frequent gene alteration in Caucasians is *KRAS* mutation, with an incidence of 30% (19). The high incidence of the *IGF2* mutation in the present study, as compared with the incidences of other genes detected in previous studies, suggests an important role for the *IGF2* mutation in lung carcinogenesis.

In the present study, *IGF2* LOI was detected in approximately half of adenocarcinomas but not in any of the squamous cell carcinomas examined; thus, *IGF2* LOI may be a marker of lung adenocarcinoma. Distinguishing squamous cell carcinoma from adenocarcinoma of the lung is important, since the therapeutic methods employed are different and molecular analysis is guided by the histology. Molecular-targeted therapeutic drugs for activated *EGFR* have resulted in improvements in response rates and progression-free survival times in lung adenocarcinoma (20).

In conclusion, in the present study, *IGF*2 LOI was observed to occur at a high frequency in lung adenocarcinoma, but was not observed in squamous cell carcinoma. This result suggests that distinct carcinogenic pathways may exist for lung adenocarcinoma and squamous cell carcinoma, depending on the *IGF2* genomic imprinting status. Therefore, *IGF2* may have potential value as a diagnostic marker and therapeutic target.

## Figures and Tables

**Table I:** IGF2 status of laser-capture-microdissected carcinoma cells, defined as imprinted or LOI in informative cases, evaluated by PCR-RFLP in combination with direct sequencing.

Case number	PCR (%T)	Sequence (%T)	Status	Histology	Differentiation of adenocarcinoma
1	0	12	Imprinted	SCC	
2	72	44	LOI	Adenocarcinoma	Well
8	54	25	LOI	Adenocarcinoma	Well
10	77	33	LOI	Adenocarcinoma	Well
11	0	17	Imprinted	SCC	
12	0	18	Imprinted	SCC	
13	62	69	LOI	Adenocarcinoma	Moderate
14	73	41	LOI	Large cell carcinoma	
15	58	46	LOI	Adenocarcinoma	Moderate
16	0	16	Imprinted	Adenocarcinoma	Moderate
17	0	16	Imprinted	SCC	
18	0	11	Imprinted	Adenocarcinoma	Moderate
19	9	17	Imprinted	Adenocarcinoma	Well
20	7	16	Imprinted	Adenocarcinoma	Moderate
21	5	16	Imprinted	SCC	
24	0	13	Imprinted	SCC	
25	0	3	Imprinted	Adenocarcinoma	Poor
26	83	62	LOI	Adenocarcinoma	Poor
28	100	85	Imprinted	SCC	
29	60	35	LOI	Adenocarcinoma	Moderate
30	0	22	Imprinted	Adenocarcinoma	Well
31	100	86	Imprinted	SCC	
32	0	9	Imprinted	SCC	

The density ratios of the top and bottom bands (bottom/top+bottom) in PCR-RFLP and the height ratios of T and C peaks (T/T+C) in sequencing are listed. When only one band was identified with PCR-RFLP (ratio=10–90%) or the sequence pattern revealed a single peak (ratio = 20–80%), the status was considered to be LOI; otherwise, the status was considered to be imprinted. IGF2, insulin-like growth factor 2; LOI, loss of imprinting; PCR-RFLP, polymerase chain reaction-restriction fragment length polymorphism; SCC, squamous cell carcinoma.
